# Implementation of a learning health system for the management of non-communicable diseases in Thailand: a realist evaluation protocol

**DOI:** 10.1136/bmjopen-2025-109261

**Published:** 2026-07-16

**Authors:** Milena Marszalek, Nutchar Wiwatkunupakarn, Nida Buawangpong, Kawinthip Rinpon, Natasha Herber, Chris Carvalho, Borislava Mihaylova, Dorothea Nitsch, Rohini Mathur, Tracey Chantler, Chaisiri Angkurawaranon, Wichuda Jiraporncharoen, John Alexander Ford

**Affiliations:** 1 Wolfson Institute of Population Health, Queen Mary University of London, London, UK; 2 Department of Family Medicine, Chiang Mai University Faculty of Medicine, Chiang Mai, Thailand; 3 London School of Hygiene & Tropical Medicine, London, UK; 4 Nuffield Department of Population Health, University of Oxford, Oxford, UK

**Keywords:** Hypertension, Digital Technology, Quality Improvement, Diabetes Mellitus, Type 2

## Abstract

**Abstract:**

**Introduction:**

Learning health systems (LHS) are an approach to translate patient data into actionable clinical insights, empower healthcare teams to drive quality improvement and reduce health inequalities. Here we present a protocol for a realist evaluation to explore what works to implement a learning health system approach in primary care settings in Thailand, for whom does it work, how, why and in what circumstances.

**Methods and analysis:**

A mixed-methods realist evaluation will run in parallel with an interventional trial [Reg No: NCT06873243] in Northern Thailand which aims to improve the management of hypertension (HTN), type 2 diabetes mellitus (T2DM) and chronic kidney diseases (CKDs) using a data-supported learning health systems approach. As part of the trial, 16 primary care units (PCUs) in Chiang Mai and Lamphun provinces will be randomly selected to receive a learning health system intervention to support quality improvement for care of HTN, T2DM and CKD. Performance will be compared between intervention PCUs and all other PCUs in the region. Participants of the realist evaluation will include clinical and other professional staff involved in the development and implementation of the LHS. This realist evaluation will use both quantitative and qualitative data, including semi-structured interviews, surveys and documents from participating sites. Quantitative and qualitative findings will be systematically integrated to test, refine and validate context-mechanism-outcomes to identify consistencies, contradictions and explanatory mechanisms as part of a final programme theory for the successful implementation of the LHS.

**Ethics and dissemination:**

Ethical approval has been granted by all collaborating university Research Ethics Committees (ref: 1090, 0321, 32540). Results will be disseminated to stakeholders, including patients and the public, health providers, the Thai government and WHO office. Our methods and dissemination will be guided by National Institute for Health Research and Guidelines International Network reporting standards for Patient and Public Involvement and Engagement.

**STRENGTHS AND LIMITATIONS OF THIS STUDY**

- A key strength is that realist methodology will allow us to develop in-depth and real-world theory about the implementation of learning health systems in settings with limited integration of electronic health records.
- This evaluation uses mixed methods and data from a broad range of stakeholders including both clinical and non-clinical staff.
- A lack of focus groups may impede social consensus about potential programme theory development.
- The quantitative analysis relies on routinely collected data which may include recording errors and missing data.
- Collecting qualitative data in busy clinical settings which are relatively research naive may limit the depth to which we can identify underlying mechanisms.

## Background

### Non-communicable disease burden in Thailand

 Non-communicable diseases (NCDs) are the leading cause of death globally, accounting for most premature mortality and long-term disability.^1^ The growing burden of cardiovascular diseases (CVDs), type 2 diabetes mellitus (T2DM), chronic kidney disease (CKD) and cancers places sustained pressure on health systems, particularly in low- and middle-income countries (LMICs).^2^

In Thailand, NCDs account for 74% of all deaths and are a major contributor to premature mortality.^3^ Hypertension (HTN) and T2DM are highly prevalent and remain suboptimally controlled despite widespread service coverage under universal health coverage operated by the National Health Security Office.^4–6^ It is estimated that reducing the number of people living with HTN in Thailand can prevent 14 000 deaths, 27 000 strokes and 18 000 heart attacks over the next 5 years.^7^

### Non-communicable disease management in Thailand

Most NCD cases in Thailand are managed in primary care units (PCUs), which function as the frontline for the district health system.^8^ PCUs are responsible for screening, diagnosis, treatment initiation, long-term follow-up and prevention activities.^8^ Care delivery in PCUs is led by multidisciplinary teams, including nurses, public health technical officers, general practitioners (GPs) and village health volunteers.^8^ Typically, each PCU has around three to five staff and is mainly operated by nurses. Most PCUs work under district hospitals, with GPs rotating to PCUs once a month on a NCD clinic day.^9^ One PCU covers a population of approximately 10 000.^8^

While this team-based model provides strong structural foundations for chronic care management, PCUs operate as complex adaptive systems. Differences in staffing, leadership, workload, digital literacy and community context create substantial variation in how care is organised and delivered, which then affects PCU clinical performance outcomes.^9–13^ Furthermore, clinical audits suggest that variation in clinical performance outcomes reflects availability of healthcare resources and varying degrees of diagnostic and treatment inertia.^9^

**Figure 1:**
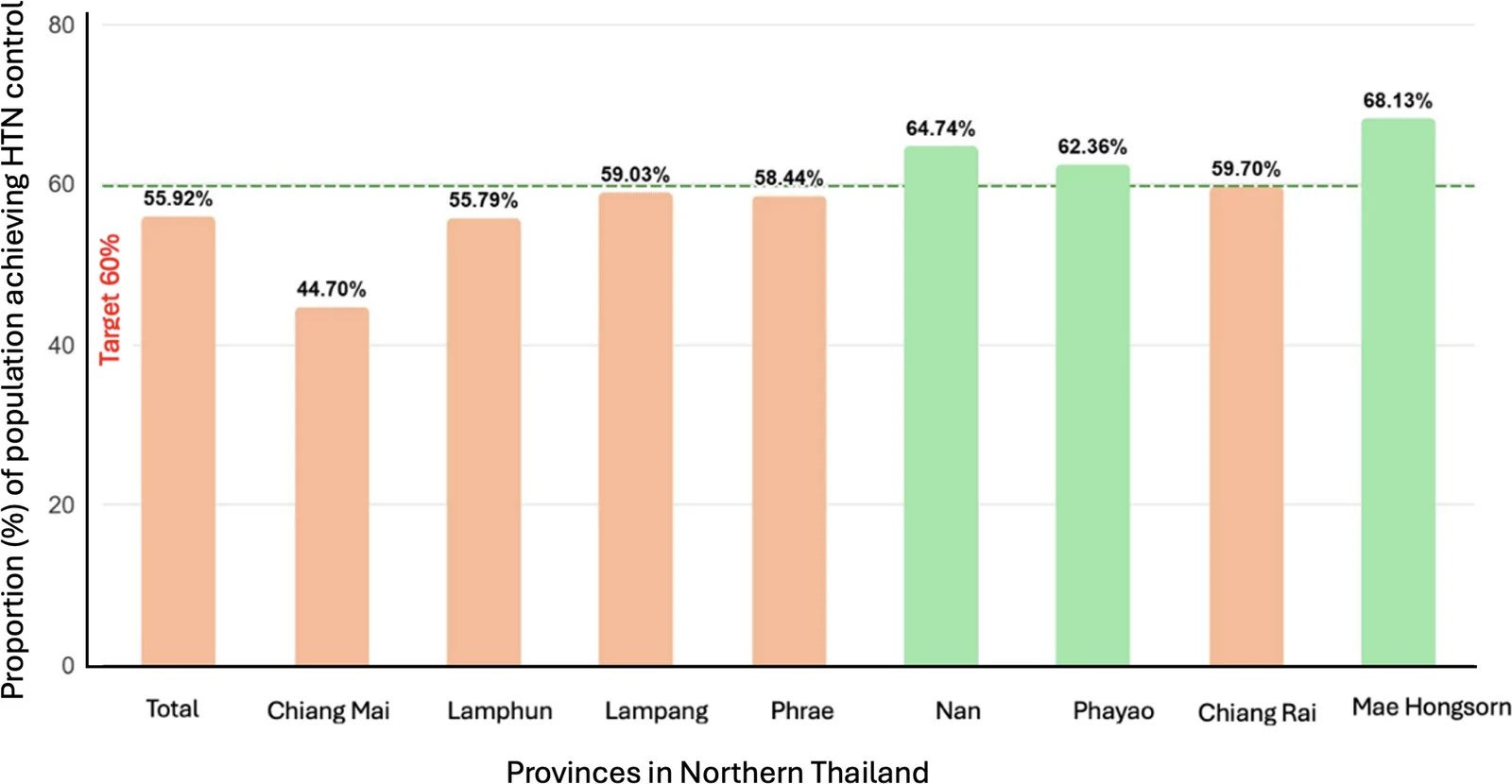
Variation in HTN control across provinces in northern Thailand.^39^ HTN, hypertension.

In Thailand, electronic health record (EHR) data is used by the Ministry of Public Health to understand how whole regions deliver care.^14^ Health data from all public healthcare facilities in Thailand is uploaded to the central government via the Health Data Centre (HDC) and visualised through the HDC online dashboard.^14^ While this national system offers an overview of population health and facility performance, data is primarily collected for administrative reporting rather than local learning.^15^ Consequently, improvements in NCD outcomes are uneven across facilities, suggesting that availability of data alone is insufficient to drive quality improvement (figure 1).

Thailand is in the early stages of using EHRs to support quality improvement for people living with NCDs.^16^ A quality improvement (QI) intervention for HTN incorporating pop-up alerts in the EHR highlighted the importance of user-friendly decision support tools to improve ease of adoption and acceptance by healthcare workers.^17^ An ongoing study across northern Thailand is trialling the implementation of a multidisciplinary QI programme to improve screening, prevention, diagnosis and management of people with CKD.^18^ The intervention is supported by computerised decision support tools which interrogate electronic patient data to create patient lists and report summary data to healthcare teams in real time.^18^

It is recognised that NCDs co-occur in a substantive number of patients, and therefore cannot be managed in isolation. Therefore, complex systems thinking is required for the development of interventions that can tackle the burden of NCDs across the heterogenous landscape of services across Thailand. One such approach is the learning health systems (LHS) concept—guiding health systems through learning using different methods such as information, deliberation and action at different systemic levels for capacity building and health system strengthening.^19^ For example, with LHS approaches, healthcare teams can leverage EHR data to provide better healthcare and reduce inequalities.^20^

In high-income countries, LHS approaches have demonstrated benefits in chronic disease management and health system performance.^21–24^ However, evidence from LMICs—particularly in primary care settings—remains limited and mixed. Similar LHS interventions implemented across different settings have produced uneven outcomes, with variation in provider engagement, sustainability and impact.^15^

Existing evaluation of LHS initiatives often focuses on measurable outcomes, such as changes in clinical indicators, while paying less attention to how contextual factors influence implementation processes.^25^ Limited attention has been given to the underlying mechanisms through which healthcare providers interpret, respond to and engage with LHS components.^25^ As a result, it remains unclear why comparable interventions function differently across primary care contexts.

Thailand is structurally well-positioned to adopt LHS approaches. The country has universal health coverage, a strong district health system and a nationwide digital health information infrastructure.^26^ However, variation in organisational culture, leadership support, analytic capacity and workflow integration across PCUs suggests that the success of an LHS intervention may depend on local contextual conditions.^8 25^

Our LHS approach and trial follow on from current QI initiatives in Thailand using EHR data to embed continuous QI into routine delivery of care for NCDs, actively building an approach that eventually will help facilitate care for people with more than one NCD.^27^ This approach has been used by the Clinical Effectiveness Group (CEG) at Queen Mary, University of London, for over 25 years.

**Figure 2:**
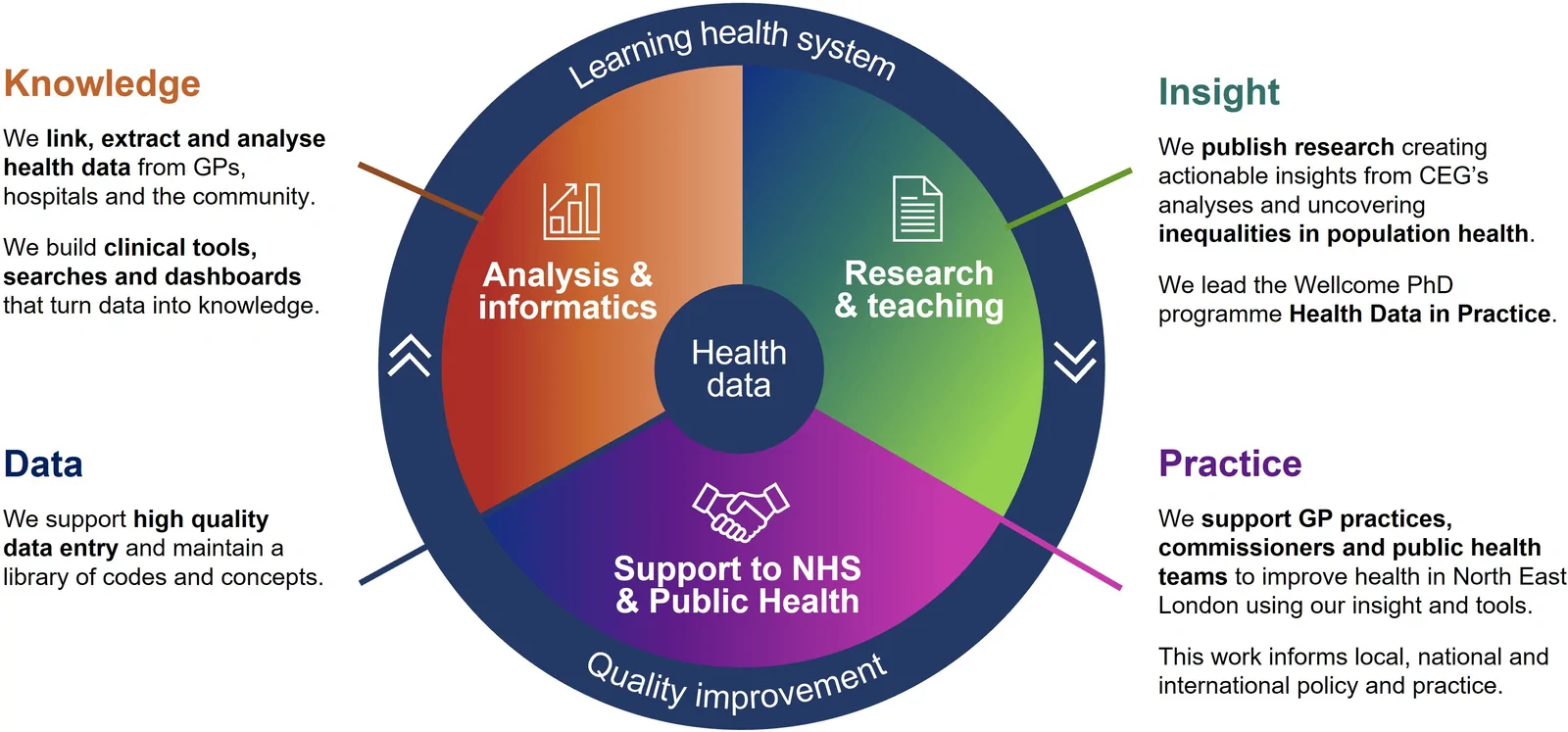
The CEG learning health system approach.^29^ CEG, Clinical Effectiveness Group; GPs, general practitioners. NHS, National Health Service

Based in north east London, CEG has implemented a clinically driven LHS in one of the most ethnically diverse and socially deprived areas of London, using locally generated data to improve the health of over 2 million people (figure 2).^28 29^ This LHS has been shown to lead to improvements in health and healthcare quality, alongside a reduction in inequalities.^30 31^

In this paper, we describe a realist evaluation to explore the individual, organisational and contextual factors and mechanisms that underpin the sustained embedding of LHS into routine delivery care for NCDs in Thailand.

### Initial programme theory

The initial programme theory (IPT) that underpins this programme has been developed based on existing evidence, experience and expert opinion of how LHSs had previously been used for NCD management in primary care in the UK and complemented by local knowledge of the Thai healthcare system. The IPT was initially drafted by the research team and then refined in a workshop with Thai stakeholders.

This IPT hypothesises how LHSs in specific contexts (C) trigger mechanisms (M) to produce outcomes (O). The initial programme theory consists of the following:

- Access to more accurate, up-to-date clinical data along with training support and collaborative learning methods across organisations provides primary care staff with the knowledge and psychological tools to drive improvements in population health.
- Through the development of a LHS that uses these elements, staff confidence in adopting digital data-driven interventions increases and data insights allow for more targeted service planning for NCD patient populations.
- The combination of these contextual and mechanistic factors will eventually lead to better management of population NCD burden, along with consistent use of improvement methodology in clinical care.
- Using collaborative learning methods will allow for the development of peer learning networks that drive further learning and innovation in practice.

This development process for this IPT began prior to the start of the clinical outcomes trial, in Jan 2025. The clinical trial commenced in July 2025, and it is currently in the stage of piloting the LHS intervention for HTN. A draft illustration of the IPT is included in online supplemental file 1.

### Evaluation questions, objectives and focus

#### Aims

The aim of our realist evaluation is to explore the impact of the LHS approach in improving NCD management in Thailand and who it works best for, in what circumstances, how and why.

#### Objectives

The specific objectives of the evaluation are to evaluate the implementation of a LHS approach for the management of HTN, T2DM and CKD in primary care in Chiang Mai and Lamphun provinces, Thailand. Furthermore, we seek to explore whether and how LHSs impact access and use of electronic health data to improve care, and to what extent this can reduce the burden of HTN, T2DM and CKD.

####  Ethical approval

Publishing our evaluation protocol aligns with UK Medical Research Council recommendations to promote methodology development and encourage study transparency. This protocol has received ethics approval from Queen Mary University of London, London School of Hygiene and Tropical Medicine (LSHTM), and Chiang Mai University (CMU) ethics boards (ref no.: 1090, 0321, 32540).

The main outcome of the evaluation will be a refined realist programme theory. In collaboration with Thai leaders in primary care and the Thai Ministry of Public Health, we will share learning and resources from this project across Thailand and globally. The Ministry of Public Health will author a national NCD service plan based on the findings of our LHS intervention. This process will be supervised by the Division of Non-communicable Diseases. The service plan will subsequently be disseminated to regional, provincial and local healthcare teams. We will produce a paper for publication in a peer-reviewed journal and present at relevant academic conferences. We will also seek to disseminate our findings through public facing and/or professional events (eg, presenting our findings at relevant hospital trusts). Funders will have no role in the decision to publish.

#### Rationale for using realist evaluation

**Figure 3:**
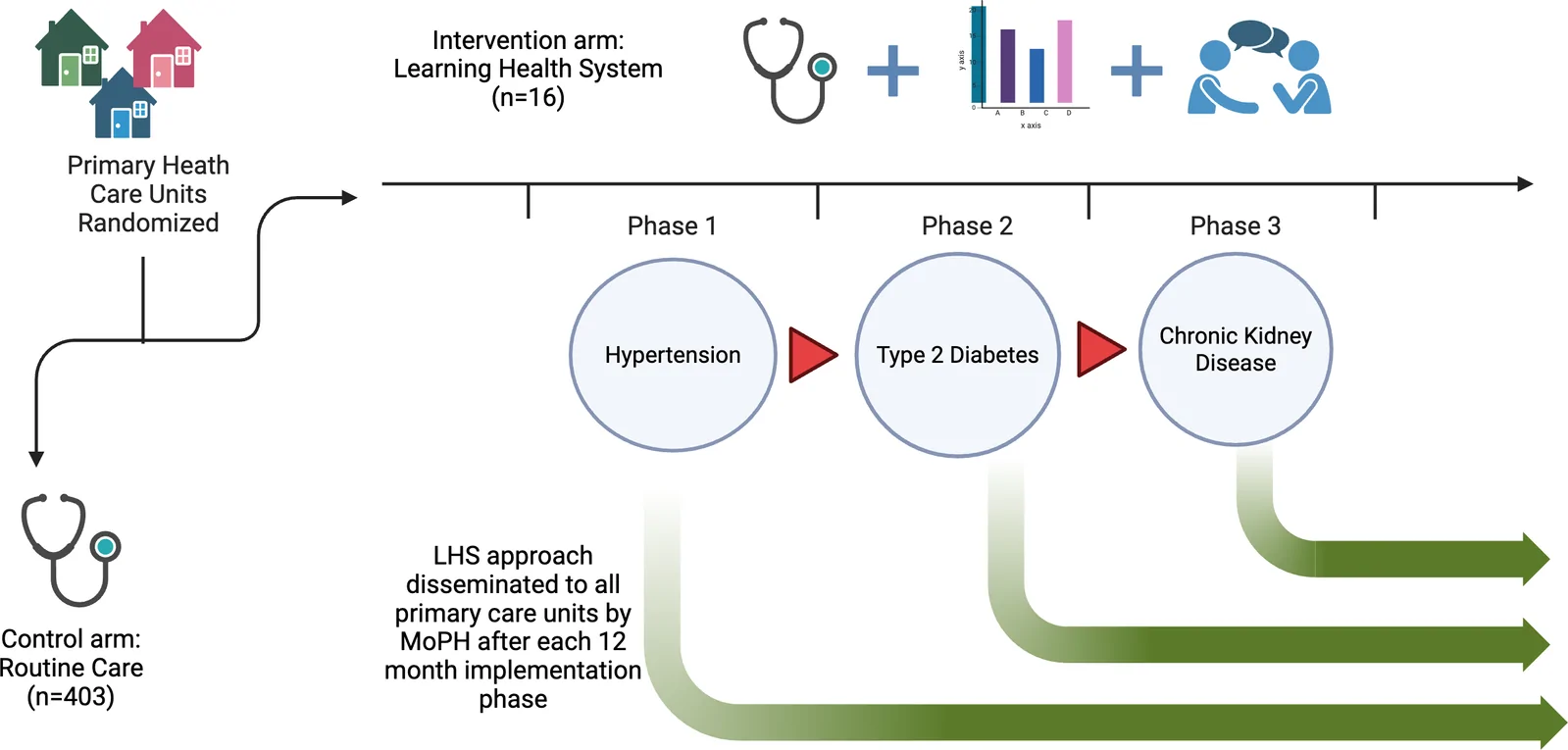
Thai LHS trial design. LHS, learning health system. MoPH, Ministry of Public Health

The LHS approach has been used internationally, with modification for differing health and care system contexts.^28^ This study will complement a LHS cluster randomised trial (RCT) and the economic evaluation that will occur in parallel. An illustration of the RCT design is included in figure 3. Random sampling has been used to allocate 16 PCUs from 419 total eligible PCUs to receive the intervention (273 units in Chiang Mai and 146 units in Lampang). This intervention is an open-label trial, as those receiving the intervention are aware of their allocation. The full details of the trial are described elsewhere.^27^ The data required for the quantitative outcomes will be collected from the EHR used in Thailand across Chiang Mai and Lamphun provinces, between 2025 and 2028, for the total population registered across the intervention PCUs for the RCT and economic evaluations.

The economic evaluation will be developed from the perspective of the Thai healthcare system and use Thai unit costs. It will include costs of staff time and other resources involved in implementation and maintenance of LHS QI programme (outside PCU staff), including development and maintenance of the Active Patient link (APL) tool, staff training, facilitation visits and other QI-related activities. In addition, PCU appointments, medications, investigations, hospital attendances and admissions of patients with target conditions will be identified using EHR data and costed. The overall additional costs, including the LHS QI programme and patient healthcare, per additional patient achieving target disease management outcome with QI programme compared with usual care will be reported.

This realist evaluation will triangulate the outcomes from the trial and economic evaluation with its own data collection. We use a realist approach because it acknowledges the importance of organisational context and seeks to understand the underpinning mechanisms, rather than make one-size-fits-all judgements.

### Intervention

**Figure 4:**
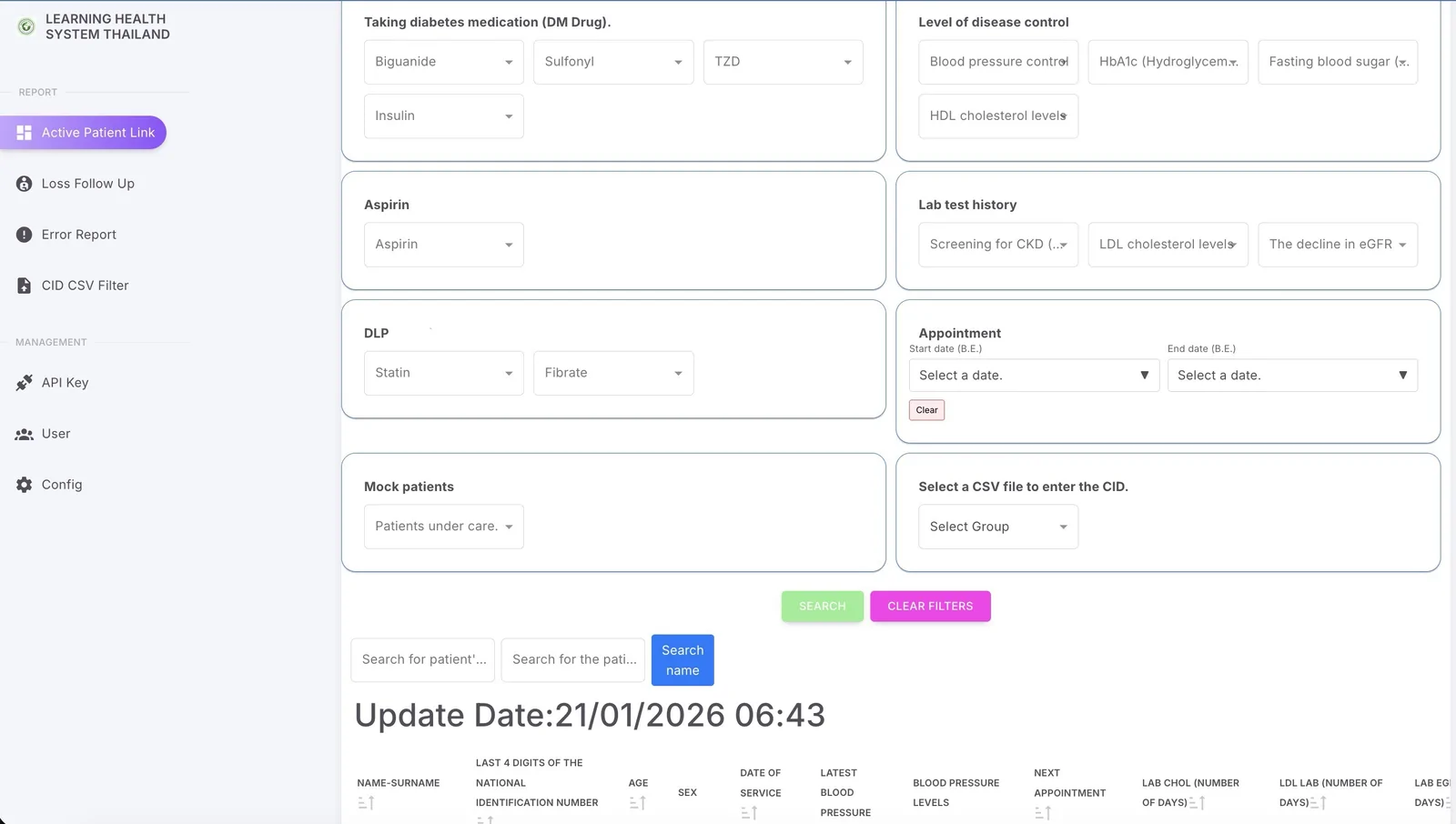
Screenshot of the APL tool for hypertension. CKD, chronic kidney disease. APL, Active Patient Link. DLP, dyslipidaemia. HDL, high-density lipoprotein. LDL, low-density lipoprotein. CID, citizen ID . eGFR, estimated glomerular filtration rate.

The LHS intervention has been based on concepts from the model of delivery used by the CEG. The LHS intervention in this context will consist of 3 elements:

1. **Data management software—the Active Patient Link tool:** to enhance data-driven quality management, the research team has developed the APL tool (figure 4)—an online platform that uses national routine EHR data to visualise actionable quality indicators for frontline healthcare workers. In addition to providing dashboards for performance monitoring, the APL tool includes a recall page that enables clinicians to identify and prioritise patients in their catchment population based on clinical risk. We will iteratively adapt the tool throughout the trial as the staff learn how to use it and will expand its use to more conditions. As many NCD patients present first with HTN, we started with HTN, but we recognise that this frequently co-occurs in people with diabetes, which is why we will focus on diabetes subsequently. Patients with diabetes and HTN are at risk of CKD, and therefore, we will then expand to CKD. As years go on, and if this approach is successful, we anticipate that staff will request add-ons for respiratory, liver and heart disease.
  At a local level, the use of the tool will vary according to clinical need. PCUs will be able to generate patient lists based on specific clinical criteria to support structured recall; they will be able to view a detailed page of information about the patient’s current medications, laboratory results and clinical observations; and they will be able to identify which patients have been lost to follow-up. The tool therefore supports proactive, individualised care planning and targeted outreach.
  The feasibility, acceptability and appropriateness of the APL tool and facilitation will be measured through the semi-structured interviews and a short survey. The survey has been devised to capture aspects of tool usability that are not covered by the interviews.
2. **Facilitation support:** to foster sustainable change and embed QI into everyday practice, structured facilitation support through both online and in-person engagements is being provided for the 16 intervention PCUs. Trained facilitators—a doctor, nurse and psychologist—will engage frontline workers in regular meetings to build capacity, offer tailored guidance and co-reflect on local data insights generated by the APL tool. These meetings will occur every 2 weeks, or within a time frame that suits the needs of the PCU. These facilitation sessions will serve multiple purposes: helping frontline staff interpret quality indicators, identify areas for improvement, plan and implement change ideas, and navigate operational challenges. This will ensure that frontline teams remain supported, engaged and empowered throughout the quality improvement cycle, reinforcing a culture of learning and accountability within primary care settings. The facilitation will remain in place for participating PCUs throughout the entirety of the study period.
3. **Education around embedding a culture of quality improvement into clinical practice:** the research team will conduct stakeholder consultation workshops with frontline healthcare workers from 16 sites over the 4-year trial period. The ‘stakeholders’ in question are nurse practitioners, public health officers, primary care physicians and governmental stakeholders such as the Ministry of Public Health and the local provincial public health office. These workshops happen once yearly, introducing the concept of a LHS, providing the research team with an understanding of routine clinical workflows, identifying gaps in the management of NCD cases and helping frontline staff collaboratively design actionable quality indicators. After a year of implementing LHS strategies, the 16 intervention sites will serve as community champions—sharing their lessons learnt, successful strategies and practical experiences with other practices across their province.
  Additionally, findings from the workshops will be reinforced during the facilitation support visits by the research team to ensure contextual understanding, build trust with local staff, validate findings from the workshops and support the co-design of tailored quality improvement strategies aligned with each site’s needs and capabilities.

## Methods

### Study design

The evaluation will use mixed methods to provide information about contexts and mechanisms that enable the testing and refinement of programme theories. Qualitative data collection will involve gathering relevant documents and conducting semi-structured interviews to complement the quantitative data and provide insights into the barriers and facilitators of implementing LHSs. Quantitative data collection will involve access to existing EHR sources used for analysis of outcomes for other studies evaluating the LHS intervention and QI activity metrics. The evaluation will be reported according to Realist and Meta-narrative Evidence Synthesis: Evolving Standards guidelines.^32^

### Semi-structured interviews

Semi-structured interviews will be conducted at each intervention site with key staff members. We will perform up to 18 interviews at 3-month to 6-month intervals throughout the study period, between 2025 and 2028. Participants will include PCU-based clinicians and administrative staff, provincial public health office staff, village health volunteers and hospital directors and managers from the intervention sites. Participants will be recruited as per local practice, through local clinical and administrative networks with CMU. Participant consent will be documented prior to interview. The interview topic guide will be informed by the IPT and will be structured to elicit data relevant to contexts (C), mechanisms (M) and outcomes (O). Interviews will gather data on how specific features of the LHS are experienced in organisational and professional contexts. Key areas will include the following:

- Contextual factors influencing implementation, including organisational culture, leadership structures, workload pressures and existing approaches to quality improvement.
- How improved access to patient-level EHR data and the APL tool interacts with local workflows and professional roles.
- Responses to training and facilitation support, including how these influence confidence, motivation, trust in data and perceived ownership.
- Perceived short- and medium-term changes in clinical practice, team dynamics and follow-up processes.

### Participant surveys

Prior to the semi-structured interviews, participants will be asked to fill in a short survey, focusing on tool feasibility, usability and appropriateness within staff daily workload. This survey will complement the content of the semi-structured interviews which will focus on wider factors related to intervention implementation. The semi-structured interview guide is included in online supplemental file 2. Tool uptake will be recorded through a user-log using HDC data tracking user log-ins.

### Documents analysis

Key local documents, such as board reports and meeting minutes, will be analysed to understand the outputs of QI activities. Eligible sites will include PCUs that carry out 6 monthly key performance indicator meetings and who have existing documentation from these meetings. Documents will be identified through existing key stakeholder networks with the CMU family medicine department. One to two documents will be analysed per intervention site, depending on their length and scope. We will also review the Public Health District office 3 monthly meeting minutes that have appropriate scope over PCU activity. These documents will provide us with a broader overview of the socio-political context in which the LHS is operating and how the LHS potentially impacts service planning for NCD patient populations.

### Quality improvement outcome and activity metrics

We will explore the main intervention outcomes per site and over time to gain an understanding of which sites had the largest and smallest improvements for different outcomes. We will collect data across three phases:

Phase 1: HTN

- The proportion of adults with HTN whose blood pressure meets target levels (<140/90 mm Hg) at 12 months, 24 months and 36 months.

Phase 2: type 2 diabetes

- The proportion of adults with T2DM whose blood glucose meets target levels (<6.5%/48 mmol/mol) at 12 and 24 months.

Phase 3: CKD

- The proportion of adults with CKD stages 1–4 who are tested for albuminuria at 12 months.

These clinical outcomes will be combined with our qualitative data findings to shed light on some of the mechanisms that could potentially lead to these outcomes: increased access to data through consistent and supported use of the LHS intervention (M) that leads to better HTN control (O).

We will also explore numbers of patients being lost to follow-up as an indirect outcome of the efficacy of the tool. These data will be analysed descriptively alongside the baseline and outcome data to quantitatively explore sites with most/least activity and largest/smallest improvements:

Phase 1: the proportion of patients with HTN with follow-up classified as:

- Complete/on-time: patients who attend appointments every 6 months for their HTN review within a 12-month period.
- Delayed: patients who have routine review appointments for HTN booked later than scheduled within a 12-month period.
- Lost: patients who are lost to follow-up without an appointment scheduled for routine HTN review within a 12-month period.

Phase 2: the proportion of patients with type 2 diabetes with follow-up classified as:

- Complete/On-time: Patients who attend appointments every 6 months for their type two diabetes review within a 12 month period.
- Delayed: Patients who have routine review appointments for their type two diabetes booked later than scheduled within a 12 month period.
- Lost: Patients who are lost to follow-up without an appointment scheduled for routine type two diabetes review within a 12 month period.

Phase 3: the proportion of patients with CKD stages 1–4 with follow-up classified as:

- Complete/on-time: patients who attend appointments every 6 months for their CKD stages 1–4 review within a 12-month period.
- Delayed: patients who have routine review appointments for their CKD stages 1–4 booked later than scheduled within a 12-month period.
- Lost: patients who are lost to follow-up without an appointment scheduled for routine CKD stages 1–4 review within a 12-month period.

The QI activity metrics that will be used within the realist evaluation include:

- Number and frequency of clinical team meetings in each intervention site.
- Frequency of scheduled review of national KPIs
- Number and professional roles of participants in these meetings.
- Number of individuals trained in using the APL tool in the context of QI and their professional roles.
- Quantitative feedback from training sessions—using a tool developed to collect feedback both for the tool development and facilitation training sessions.
- Total number of datasets used for QI projects.
- Volume of missing data within these datasets.

Data collection methods for these metrics will be driven by local practice around documentation of team activity—usually this is in the form of 6 monthly meetings with the provincial public health office. Baseline data for chosen metrics will be compared with data collected after a minimum of 3 months. This data will support the development of potential mechanisms as part of the programme theory; helping to triangulate service planning and quality improvement activities with staff perceptions of the LHS intervention.

### Data analysis

Quantitative data will help identify where outcomes occur, while qualitative data will explain how and why these outcomes are achieved, providing a richer understanding of the relationships between contexts, mechanisms, and outcomes. This mixed methods approach will involve:

- Comparing data types—quantitative patterns will be triangulated with qualitative insights to identify consistencies, contradictions and explanatory mechanisms.
- Iterative refinement of context-mechanism-outcome (CMO)—quantitative data will help identify where outcomes occur, while qualitative data will explain how and why these outcomes are achieved, providing a richer understanding of the relationships between contexts, mechanisms, and outcomes.

Where possible we will analyse mixed methods data concurrently to allow thematic sampling of themes within the interviews. Using the CMOs we will develop a final programme theory.

Interview data and documents will be coded using a realist logic of analysis. A reflexive diary will be kept during the qualitative analysis. Methodological rigour and validity will be maintained by having 10% of transcripts independently coded by another researcher. The qualitative data will be examined for CMO configurations, with a particular focus on the (C) and (M): does improved access to data and facilitation support within a collaborative environment (C) provide staff with the tools to adopt data-driven interventions and embed quality improvement culture in their daily clinical care (M)?

Our quantitative data analysis will include outcomes relating to improvements in population NCD burden and consistent use of improvement methodology in clinical care. An initial exploratory review of whether QI activities form a part of the PCU workload will be carried out. We will then perform a baseline analysis both for PCU demographics and their baseline QI activity, to be able to measure change in the outcomes.

### Patient and public involvement

The realist evaluation workstream will involve patient and public involvement (PPI) for both patients and staff. We will conduct PPI workshops throughout the implementation and evaluation process to test the feasibility and acceptability of the LHS for both populations. Our methods and dissemination will be guided by UK National Institute for Health Research and Guidelines International Network reporting standards for PPI.^33 34^

We have also had substantial input from our other stakeholders during the development of the intervention: the Thai Ministry of Public Health (at national, provincial and district levels), the Thai WHO and the Thai Primary Care Support Office. This group of stakeholders has provided input into both the scientific and practical aspects of implementing the LHS intervention.

Sourcing from each of the intervention units, the CMU and Queen Mary University of London (QMUL) teams will train a network of clinical and community champions who will support the intervention primary care units to deliver the LHS and engage with patients and the public. Champions will be given clear role descriptions of what patient engagement is in the context of quality improvement. We will co-produce locally relevant educational materials and guidelines for healthcare providers and patients.

### Strengths, limitations and future directions

Using a mixed-methods approach for this realist evaluation to complement the findings from the trial will give a rich understanding of the mechanistic and contextual factors contributing to trial outcomes. This evaluation will provide specific as well as generic learning about the adoption of data-enabled innovative tools and their use in practice for health improvement in an LMIC setting. The focus on both robust quantitative and qualitative methodology will give insight into how human and organisational factors contribute to changes in NCD management and can be sustained in practice. Other strengths of our study include previous CMU collaboration with the Thai Ministry of Public Health, LSHTM and the WHO to lead work integrating electronic patient records into care pathways for NCDs.^3 35^ CMU has access to pseudonymised patient level EHRs for all individuals residing in Lampang and Chiang Mai provinces—two of the largest provinces in northern Thailand and cover a wide range of rural and urban contexts, therefore improving generalisability of results.

Limitations include the possibility of a reporting bias or observation bias where mechanisms are partially shaped by the presence of the research team. The quantitative analysis relies on routinely collected data which may include recording errors and missing data. The qualitative analysis will identify underlying mechanisms, but is unlikely to be fully understood due to cultural factors around communication, hierarchy and external evaluation, particularly in the context of a UK-Thai research collaboration. This may limit the extent to which problems or concerns are reported by staff.

### Comparison with existing literature

The LHS approach has been internationally recognised as a vital part of driving better health outcomes; however, there is a continuous need to build up the evidence base about how to help LMICs use this approach in an effective way.^28^ Our study has been set up to develop and pilot a LHS intervention that already has an evidence base of success in the UK.^36^ It is novel in its approaches of using existing methods and contextualising them in an LMIC setting with differing cultural norms and working patterns.

The LHS approach acts on learning from previous community interventions aimed at improving NCD management. The CMU team have recently implemented pilot programmes in Lampang province to improve the management of HTN and diabetes in primary care.^37 38^ Interventions included (1) co-design of simple education programmes and treatment protocols, (2) training of 53 primary care teams comprising >600 people, (3) monitoring of outcomes and (4) process evaluation using EHRs. The programme improved control of HTN and diabetes, improved access to treatment and care by empowering nurses and pharmacists to prescribe treatment, reduced variation between practices and reduced treatment inertia.^35^ Furthermore, the treatment protocols developed for the interventions were endorsed by the Thai Ministry of Public Health.^38^

Despite the success of the pilot programmes on several fronts, the new approaches were not continued, largely due to lack of knowledge around how to translate the approach for use in routine clinical practice over the longer term. This paper describes the design of a mixed-methods realist evaluation that aims to understand the contextual factors leading to successful implementation of a LHS intervention.

## Supplementary material

10.1136/bmjopen-2025-109261 online supplemental file 1

10.1136/bmjopen-2025-109261 online supplemental file 2
