# Supplementary figures and images for: Implementation of a learning health system for the management of non-communicable diseases in Thailand: a realist evaluation protocol
Source: BMJ Open. 2026 Jul 16;16(7):e109261. doi: 10.1136/bmjopen-2025-109261 (PMC13384129; doi:10.1136/bmjopen-2025-109261)

Supplementary File 1.

**Draft Initial Programme Theory**


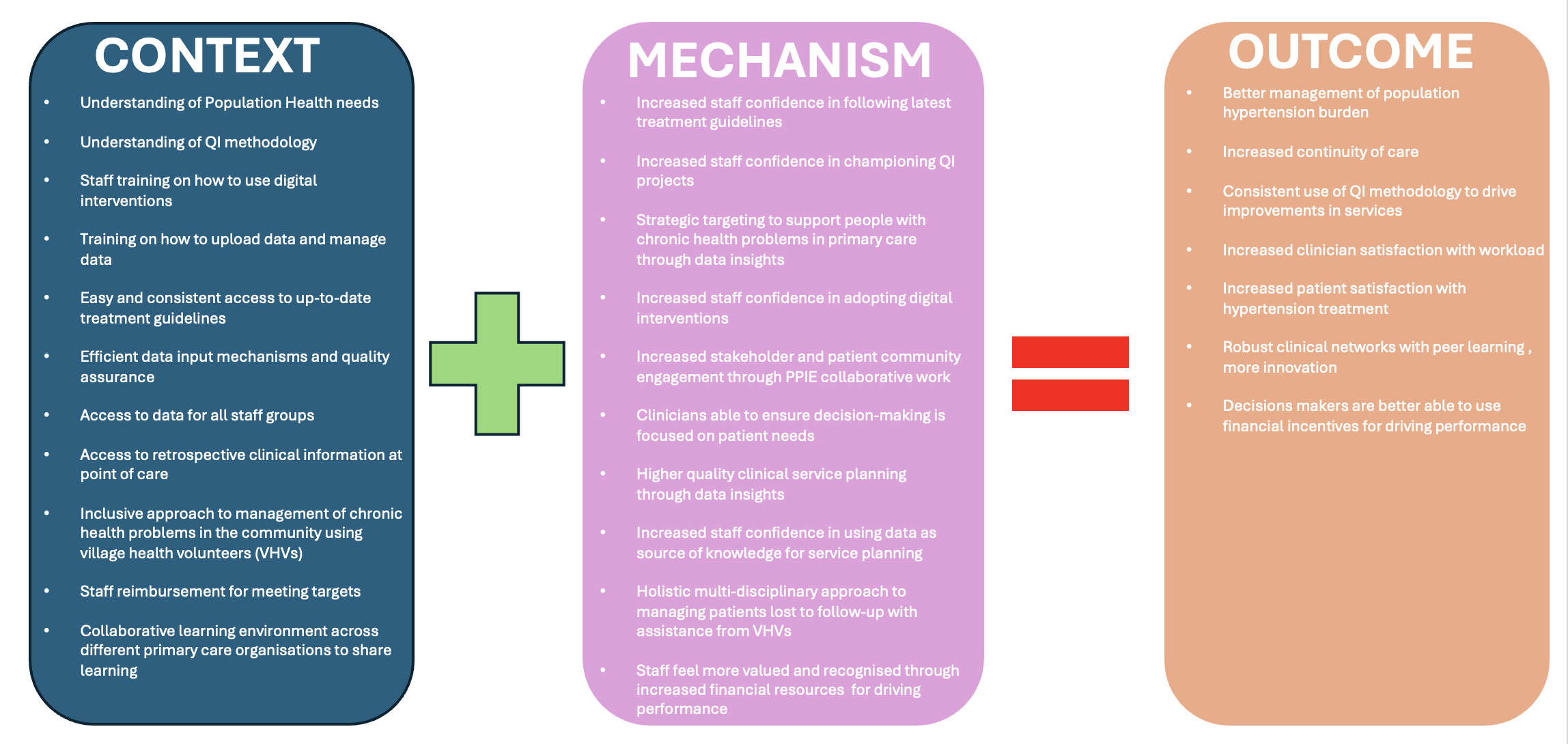

Supplement: online supplemental file 1 [file bmjopen-16-7-s001.docx]
