# Supplementary material for: Implementation of a learning health system for the management of non-communicable diseases in Thailand: a realist evaluation protocol
Source: BMJ Open. 2026 Jul 16;16(7):e109261. doi: 10.1136/bmjopen-2025-109261 (PMC13384129; doi:10.1136/bmjopen-2025-109261)
Supplement: online supplemental file 2 [file bmjopen-16-7-s002.docx]

**Supplementary File 2.**

**Draft healthcare professionals' perspectives on the use of APL (Technology Adoption Model – TAM) tools**

Statement (for respondents)

The purpose of this survey is to learn about your views on the ease of use and acceptability of the APL tool in practice. Your answers will be kept confidential and used for research purposes only. There is no right or wrong answer. Please complete the questionnaire based on your actual experience of using it.

**Part 1: Basic Information**

1. Position:

☐ Doctor
☐ Nurse
☐ Public Health officer
☐ Other (please specify): _______

1. Time spent in current role
   ☐ < 5 years
   ☐ 5–10 years
   ☐ > 10 years
2. Role in caring for patients with hypertension (choose more than one if necessary)

☐ Screening
 ☐ Diagnostics

☐ Treatment
 ☐ Monitoring

☐ Referral
 ☐ Health Promotion and Disease Prevention

☐ Others (please specify): _____________________

1. Please rate your level of confidence in your technology skills.

Where 10 = Most confident 1 = I'm not confident at all.

1 2 3 4 5 6 7 8 9 10

1. How long have you been using the APL tool?

< 1 month ☐

1–3 months ☐

> 3 months ☐

**Scoring Criteria**

For sections 2-6, please provide an answer that matches your opinion.

1 = Strongly disagree

2 = Disagree

3 = Neutral

4 = Agree

5 = Strongly agree

**Part 2: Perceiving the benefits**

*Healthcare professionals' level of belief in the use of APL tools to increase the efficiency of hypertension management and patient care quality.*

| List | 1  Strongly disagree | 2  Disagree | 3  Neutral | 4  Agree | 5  Strongly agree. |
| --- | --- | --- | --- | --- | --- |
| 1. Using the APL tool allows me to view a list of patients who need to be followed up at my facility. |  |  |  |  |  |
| 1. The APL tool makes it easier for me to access patient information in my electronic medical records. |  |  |  |  |  |
| 1. The APL tool helps me track patients more efficiently. |  |  |  |  |  |
| 1. Using the APL tool helps me to prioritize my patients' problems. |  |  |  |  |  |
| 1. The APL tool provides enough information for me to care for hypertensive patients. |  |  |  |  |  |

**Part 3: Perceived ease of use**

*The level of belief among medical professionals is that the APL tool is easy to use.*

| List | 1  Strongly disagree | 2  Disagree | 3  Neutral | 4  Agree | 5  Strongly agree. |
| --- | --- | --- | --- | --- | --- |
| 1. Learning to use the APL tool was easy for me. |  |  |  |  |  |
| 1. Using the APL tool in my daily patient care is easy. |  |  |  |  |  |
| 1. The process of using the APL tool is clear and easy to understand. |  |  |  |  |  |
| 1. I can operate the APL tool without additional technical assistance. |  |  |  |  |  |
| 1. Practicing using the APL tool until I became proficient was not difficult for me. |  |  |  |  |  |

**Part 4: Attitude to Use**

*Healthcare Provider's overall positive or negative sentiment level towards the use of APL tools*

| List | 1  Strongly disagree. | 2  Disagree | 3  Neutral | 4  Agree | 5  Strongly agree. |
| --- | --- | --- | --- | --- | --- |
| 1. I think the introduction of the APL tool in clinical practice is a good idea. |  |  |  |  |  |
| 1. I have a good feeling about using the APL tool. |  |  |  |  |  |
| 1. I like to use the APL tool as part of my work. |  |  |  |  |  |

**Part 5: Behavioral Intention to Use**

*The level of intention of healthcare professionals to continue using the APL tool*

| List | 1  Strongly disagree | 2  Disagree | 3  Neutral | 4  Agree | 5  Strongly agree. |
| --- | --- | --- | --- | --- | --- |
| 1. I intend to use the APL tool consistently in my work |  |  |  |  |  |
| 1. I will continue to use the APL tool to care for patients with hypertension. Diabetes and chronic kidney disease |  |  |  |  |  |
| 1. I would recommend the APL tool to other healthcare professionals. |  |  |  |  |  |

**Part 6: Miscellaneous**

| List | 1  Strongly disagree. | 2  Disagree | 3  Neutral | 4  Agree | 5  Strongly agree. |
| --- | --- | --- | --- | --- | --- |
| 1. I think the purpose of the APL tool for hypertension is clear. |  |  |  |  |  |
| 1. I think the APL tool manual made by the research team made it easier for me to use the tool. |  |  |  |  |  |
| 1. I have received adequate training from the research team to use the APL tool effectively. |  |  |  |  |  |
| 1. I have received adequate support to be able to use the APL tool effectively through the following:   -obtaining an account to access the service  -having internet at my facility to connect to the APL tool,  -a computer and adequate power to access the APL tool |  |  |  |  |  |

**Draft Semi Structured Interview Template: LHS Realist Evaluation**

**Introduction: Hello** , my name is ………. And I am part of the team that is evaluating the LHS project for NCDs. I am not part of the implementation team.

**Purpose of the interview**

The purpose of this interview is to explore your experience related to the Learning Health System, including the APL Tool, the APL Tool Manual, the follow-up by the facilitation team, as well as the workshops to improve the quality of services for Patients with non-communicable diseases.

**Detail**

This interview will last about 45-60 minutes. If you wish to suspend or terminate the interview at any time, please inform the interviewer at any time. Information you provided in this interview will be kept confidential. No personally identifiable information is disclosed throughout the research process.

**Consent**

Before the interview, the investigator will check:

-That you have read the participant information sheet in full.

- That all your questions have been answered

- That the written consent form has been signed.

**Opening question:**

**Please tell us about your experience of using the APL Tool.**

- Additional questions: Which areas did you find most useful? Which functions were not used in the APL Tool?
- Can you explain a bit more about how easy you found the tool to use (see survey section 3).
- What is your understanding of what the APL tool is used for?

(e.g., assisting during patient consultations, or prioritizing all patients in the service unit)

- Please tell us more about how you use the APL tool in your daily work.
- Who are the main users in your PCU or hospital? Why do you think they are the main users?

**Which groups of patients have you used this tool with? / Which group benefits the most? Which groups have benefited less and why?**

- Additional Questions: What is your role of caring for NCD patients with complications in collaboration with the hospital?

**Please tell us about your experience of attending the workshop and the support of the facilitation team, including the APL Tool manual that you have received?**

- Additional questions: Please tell us about your experience using APL Tool-like programs in your unit and how it went.

**If you have a list from the APL Tool, do you have any obstacles in continuing to use the APL Tool ? Can you tell me more about them?**

- More questions: is the workload excessive?

**How do you think this project has impacted your workload and made a difference to care for hypertension patients?**

- How has your service unit changed the tools and training contributing to the implementation of quality development?
- How does it work with your existing workflows?

**The whole project aims to improve the care of patients with hypertension.**

- Can you tell me about how this project helps you solve any problems that arise in caring for patients with hypertension In your hospital/hospital?

**Tell us about your experience in exchanging learning from this project with other service units from the past workshops.**

**What are your opinions about how the project affects the work of the team and colleagues in your PCU?**

- For example, working with the village health volunteers, in the community or multidisciplinary team.

**What are your suggestions for improving or developing this tool to improve long-term performance (see questionnaire, section 5)?**

- Additional question: Do you think you will continue to use this tool in the long term? Yes or no, why?

Thank you for taking the time to participate in this interview.
