# Supplementary material for: Implementation of a learning health system for the management of non-communicable diseases in Thailand: a realist evaluation protocol
Source: BMJ Open. 2026 Jul 16;16(7):e109261. doi: 10.1136/bmjopen-2025-109261 (PMC13384129; doi:10.1136/bmjopen-2025-109261)
Supplement: Supplementary file 3 [file bmjopen-2025-109261.reviewer_comments.pdf]

PEER REVIEW HISTORY

BMJ Open publishes all reviews undertaken for accepted manuscripts. Reviewers are asked to complete a checklist review form and are provided with free text boxes to elaborate on their assessment. These free text comments are reproduced below.

ARTICLE DETAILS

Title (Provisional)

Implementation of a learning health system for the management of non-communicable diseases in Thailand: A realist evaluation protocol

Authors

Marszalek, Milena; Wiwatkunupakarn, Nutchar; Buawangpong, Nida; Rinpon, Kawinthip; Herber, Natasha; Carvalho, Chris; Mihaylova, Borislava; Nitsch, Dorothea; Mathur, Rohini; Chantler, Tracey; Angkurawaranon, Chaisiri; Jiraporncharoen, Wichuda; Ford, John Alexander

---

VERSION 1 - REVIEW

**Reviewer**	**1**
**Name**	**Hebbar, Pragati**
**Affiliation**	**Maastricht University**
**Date**	**04-Jan-2026**
**COI**	**None**

An interesting, timely and relevant study. After going through the submission there seem to be some areas that need to be clarified to help the reader. I have listed down the points in a separate document. It appears like the initial program theory has been developed, in which case it would be good to add it to this paper.

BMJOpen review comments:

Overall an important contribution to the field. Below please find some overarching and more specific comments on the manuscript

- In the abstract, the introduction section can be reworked towards the end to deepen the understanding related to realist evaluation. The current framing of the last sentence is not adequately communicating the gist of realist approach.
- In the strengths and limitation section on page 5 the last two points on the FGDs and missing quantitative data seem like limitations that could have been addressed during the study design
- The text in Figure 1 in the title, legend as well as names of all the provinces are not in English and difficult for an audience following English language to interpret.

- Page 7, lines 37 to 48 mentioning the disease burden needs to be cited appropriately
- The figure 2 is grainy and doesn't seem to be originally designed for this paper. The original source is not cited if this is being reproduced.
- Page 10, line 27, an abbreviation CMU is used for the first time and not expanded.
- Figure 4 is a screenshot and again grainy and with lot of non-English text some explanation of the figure would help explain better
- The theoretical framework sub-heading seems repetitive and a bit redundant after the content of the study design section above it. Consider altering to avoid repetition or dropping it. If there are any other theories/ frameworks guiding the realist evaluation they can be added here.
- The initial program theory section explains the different sources of data; can the authors correct the tense of the sentences as some are future tense while some are past tense in framing. It is not clear if the IPT development is ongoing or completed. Since it is mentioned that the IPT will be developed from evidence, experience and expert opinion and refined through a workshop. UK team has provided online support for this workshop. If this is completed, then it is important to include the IPT into this protocol paper.
- Under the objectives an abbreviation 'LTC' is found which is not found anywhere else in the paper. Please expand
- The aim and objective need to be reworked as currently the aim is more specific whereas the specific objectives are too broad and not specific or measurable.
- Page 17 mentions activity metrics for realist process evaluation. These supplementary measures might help test and refine the program theories. Unlike process evaluation which usually explains the fidelity of the implementation here the intent of using a realist evaluation is different, which is to explain mechanisms. Re-look and revise this segment and avoid the term 'realist process evaluation'
- Page 20, line 24 what is meant by PPIE, please expand it on page 4, line 22 where it is mentioned for the first time
- The list of references at the end needs to be checked and revised, some references have two or more full stops at the end while some have none. Ensure it follows the journals referencing guidelines and requirements
- The interview guide has been shared and has some typographical error on page 38. Also without an initial program theory looking at an interview guide might not be very useful as the data collection needs to be guided by the IPT.
- In the third ICMJE declaration form the name of the person is missing

---

**Reviewer**	**2**
**Name**	**Zurynski, Yvonne**
**Affiliation**	**Macquarie University, Australian Institute of Health Innnovation**
**Date**	**29-Jan-2026**

COI None

Thank you for the opportunity to review this potentially interesting paper. However, I feel the authors need to undertake a thorough review and resubmit it, before it can be considered for publication.

I found the manuscript vague in parts and not structured according to the RAMESES II guidelines (Wong et al. 2016). The rationale for a realist evaluation needs to be clarified and strengthened and the evaluation questions must be more clearly articulated.

There is no description of the context, leaving the reader wondering what the Thai Primary Care Units are, how they are staffed and funded and what services they deliver and their level of experience with electronic medical records at baseline. This is especially important given that methods tested in the UK are being applied to a very different context. A description of the participating PCUs and the contexts they operate in would be helpful – a fundamental consideration for any evaluation, especially a realist evaluation.

The details of the intervention were also a little hard to follow – what exactly is being implemented and how? Stating that the trial will implement a culture of quality improvement through "stakeholder consultation workshops" is a little misguided – culture change requires time and sustained effort. There was no information about how many stakeholder workshops will be held, over what period of time and with which stakeholders exactly?

A more detailed description of the APL tool and how it is intended to be used at the local levels is required. Information on how you will measure acceptability, appropriateness, uptake and feasibility of the APL tool among staff in the PCUs has not been described.

It's hard to follow the narrative of the manuscript at times. The rationale for selecting the three conditions needs to be much clearer. The conditions are variously described as heart disease, hypertension, HTN, strokes, heart attacks… although it appears (but is not clearly articulated that the that the target is hypertension). There is a clear overlap between cardiovascular disease and type-2 diabetes but thisoverlap is not addressed – how will data from patients who have both conditions be treated?

The outcomes provided on p16 of 23 report clinical improvements – in a realist evaluation I would have expected (for example) an exploration of the mechanisms that led to an increased proportion of adults with hypertension reaching their blood pressure targets rather than simply reporting the proportions. It is not at all clear whether this will be done and how?

The statement about patients being lost to follow-up as an indirect outcome of the efficacy of the tool needs greater explanation (p16 of 63)… again the reasons or mechanisms for the number of on-time, delayed or lost to follow-up would align with a realist evaluation – how will the reasons or mechanisms be identified and defined through the proposed work?

Please provide references for all statements quoting statistics e.g. paragraph 2, p7 of 63.

The terminology used throughout the manuscript is confusing and inconsistent e.g. diabetes (p7 of 63), then we have T2DM used for the first time without being defined (p9 or 63) and then we move to Type 2 Diabetes (p16 of 63).

The many acronyms must be consistently defined in the text before being used and once defined must be used consistently – there are too many of these to enumerate, e.g. NIHR, G-I-N and PPIE in the abstract; PPIE, QMUL, CMU, HTN and many others in the in manuscript.

I have concerns regarding statements such as "...reach absolute truth" - what is meant by this? How do you define "absolute truth"? (p5 of 63)

The authors must clarify what work has already been undertaken (e.g. to develop an initial program theory) and what is being proposed in this protocol – there appears to be considerable overlap. Has the clinical outcomes trial already begun and if so, what stage has been reached?

Greater clarity is needed with regards to the proposed methods e.g. what will you do and how, to identify the CMOs – the outcomes are described but the methods for identifying and refining contexts and mechanisms need to be more clearly described and logically organised to adhere to the RAMESES II reporting guidelines and realist methodologies and approaches.

Please describe the methods more precisely – for example: how many consultations will be performed, with which stakeholders and at what time points in the implementation. How exactly will the information from consultations be used to develop the CMOs? What validation will be undertaken with whom and by what means?

What is the definition of urbanity? How will it be measured? What are the categories - is it just urban and rural? – how are these defined in the Thai context?

An economic evaluation is mentioned without providing any details (p12 of 63). Please clearly articulate the relevance of the economic evaluation to the realist evaluation.

There is an assertion that "programmes achieved success… " however, no evidence has been provided of this success (p22 of 63).

Figures and tables:

Some of the figures are illegible e.g. Figure 4;

One figure has captions in Thai script – this is not appropriate for an international audience; The figures need footnotes to define the many acronyms used.

Do the authors have permissions to reproduce Figure 2 -the figure seems to have been copied an existing publication.

It's not clear who "commissioners" refers to in Figure 2.

References:

The references are not consistently formatted (especially references to online documents or websites) and there are a number of other problems e.g. why are you referring to a pre-print from 2022? (ref 5) – either refer to the published paper or choose another reference, there are incomplete references e.g. ref 18.

VERSION 1 - AUTHOR RESPONSE

Reviewers comments	Response
Reviewer 1: An interesting, timely and relevant study. After going through the submission there seem to be some areas that need to be clarified to help the reader. I have listed down the points in a separate document. It appears like the initial program theory has been developed, in which case it would be good to add it to this paper.	We are grateful to reviewer 1 for their review. We address all points below.<br>Add in as supplementary figure.
In the abstract, the introduction section can be reworked towards the end to deepen the understanding related to realist evaluation. The current framing of the last sentence is not adequately communicating the gist of realist approach.	Agree. We have revised the abstract to make align more with realist approaches
In the strengths and limitation section on page 5 the last two points on the FGDs and missing quantitative data seem like limitations that could have been addressed during the study design	Agree. We have revised the strengths and limitations section to highlight the potential limitations from the qualitative and quantitative data and analysis.
The text in Figure 1 in the title, legend as well as names of all the provinces are not in English and difficult for an audience following English language to interpret.	This figure has been replaced with a descriptive analysis of HTN control across Northern provinces only, with English labels.
Page 7, lines 37 to 48 mentioning the disease burden needs to be cited appropriately	These figures derive from internal data at the Thai Ministry of Public Health and we have clarified that in the text.
The figure 2 is grainy and doesn't seem to be originally designed for this paper. The original source is not cited if this is being reproduced.	This image has now been replaced with a higher quality image. This image represents the ethos of working of the Clinical Effectiveness Group- the design of the LHS is based on this ethos.
Page 10, line 27, an abbreviation CMU is used for the first time and not expanded.	Now spelt out, thank you for spotting that.
Figure 4 is a screenshot and again grainy and with lot of non-English text some explanation of the figure would help explain better	Figure 4 has been replaced.
The theoretical framework sub-heading seems repetitive and a bit redundant after the content of the study design section above it. Consider altering to avoid repetition or dropping it. If there are any other theories/ frameworks guiding the realist evaluation they can be added here.	Removed

The initial program theory section explains the different sources of data; can the authors correct the tense of the sentences as some are future tense while some are past tense in framing. It is not clear if the IPT development is ongoing or completed. Since it is mentioned that the IPT will be developed from evidence, experience and expert opinion and refined through a workshop. UK team has provided online support for this workshop. If this is completed, then it is important to include the IPT into this protocol paper.	We have now included further details of the IPT development. We have included high level details of the IPT and added the full IPT to a supplementary appendix.
Under the objectives an abbreviation 'LTC' is found which is not found anywhere else in the paper. Please expand	Removed.
The aim and objective need to be reworked as currently the aim is more specific whereas the specific objectives are too broad and not specific or measurable.	We have revised the aims and objectives section to ensure that they align.
Page 17 mentions activity metrics for realist process evaluation. These supplementary measures might help test and refine the program theories. Unlike process evaluation which usually explains the fidelity of the implementation here the intent of using a realist evaluation is different, which is to explain mechanisms. Re-look and revise this segment and avoid the term 'realist process evaluation'	Agree. We have revised this section, along with the outcomes section, to more accurately describe the activity metrics. We have also clarified the analysis section to ensure consistency.
Page 20, line 24 what is meant by PPIE, please expand it on page 4, line 22 where it is mentioned for the first time	Abbreviation expanded.
The list of references at the end needs to be checked and revised, some references have two or more full stops at the end while some have none. Ensure it follows the journals referencing guidelines and requirements	The references have been corrected as requested.
The interview guide has been shared and has some typographical error on page 38. Also without an initial program theory looking at an interview guide might not be very useful as the data collection needs to be guided by the IPT.	We have included a draft topic guide, however we anticipate thematic sampling within the topic guide. Therefore the exact questions will evolve as we analyse data to allow sampling of realist theory.
In the third ICMJE declaration form the name of the person is missing	
Reviewer 2: Thank you for the opportunity to review this potentially interesting paper. However, I feel the authors need to undertake a thorough review and resubmit it, before it can be considered for publication. I found the manuscript vague in parts and not structured according to the RAMESES II guidelines (Wong et al. 2016). The rationale for a realist evaluation needs to be clarified and strengthened and the evaluation questions must be more clearly articulated.	We have undertaken a major revision of the article to improve the clarity and flow. We have reviewed the RAMESES checklist and ensured that the relevant components are included.
There is no description of the context, leaving the reader wondering what the Thai Primary Care Units are, how they are staffed and funded and what services they deliver and their level of experience with electronic medical records at baseline. This is especially important given that methods tested in the UK are being applied to a very different context. A description of the participating PCUs and the contexts they operate in	Context section added.

would be helpful – a fundamental consideration for any evaluation, especially a realist evaluation.	
The details of the intervention were also a little hard to follow – what exactly is being implemented and how? Stating that the trial will implement a culture of quality improvement through "stakeholder consultation workshops" is a little misguided – culture change requires time and sustained effort. There was no information about how many stakeholder workshops will be held, over what period of time and with which stakeholders exactly?	We have added details on how the intervention works in the respective section. The key aspect is that health care workers are not just shown a dashboard, but instead can access patient lists as to who needs to be seen to improve the dashboard metric.<br>Regarding the stakeholder workshops, we have confirmed the detail about what the consultations will involve and how they relate to the main data collection methods for the realist evaluation.
A more detailed description of the APL tool and how it is intended to be used at the local levels is required. Information on how you will measure acceptability, appropriateness, uptake and feasibility of the APL tool among staff in the PCUs has not been described.	Feasibility will be assessed within the semi-structured interviews. Appropriateness and acceptability will be assessed both using a short survey and the semi-structured interviews. Tool uptake will be recorded through a user-log using HDC log-in data.
It's hard to follow the narrative of the manuscript at times. The rationale for selecting the three conditions needs to be much clearer. The conditions are variously described as heart disease, hypertension, HTN, strokes, heart attacks… although it appears (but is not clearly articulated that the that the target is hypertension). There is a clear overlap between cardiovascular disease and type-2 diabetes but thisoverlap is not addressed – how will data from patients who have both conditions be treated?	We have clarified now at the very start of the manuscript for those who are not clinicians that these conditions frequently co-occur (as most NCDs do, in fact, because of shared underlying risk factors). This therefore requires a non-vertical approach, and a tool that can adapt to more than one condition. In the trial, we are starting with hypertension, then expand to diabetes, then to CKD to make it easier for staff to learn how the LHS approach works.
The outcomes provided on p16 of 23 report clinical improvements – in a realist evaluation I would have expected (for example) an exploration of the mechanisms that led to an increased proportion of adults with hypertension reaching their blood pressure targets rather than simply reporting the proportions. It is not at all clear whether this will be done and how?	We have revised the structure and content of the outcomes/activity section and analysis section of the protocol to improve the clarity and accuracy.
The statement about patients being lost to follow-up as an indirect outcome of the efficacy of the tool needs greater explanation (p16 of 63)… again the reasons or mechanisms for the number of on-time, delayed or lost to follow-up would align with a realist evaluation – how will the reasons or mechanisms be identified and defined through the proposed work?	Have included section on this in data analysis.
Please provide references for all statements quoting statistics e.g. paragraph 2, p7 of 63.	To do.
The terminology used throughout the manuscript is confusing and inconsistent e.g. diabetes (p7 of 63), then we have T2DM used for the first time without being defined (p9 or 63) and then we move to Type 2 Diabetes (p16 of 63).	Corrected.

The many acronyms must be consistently defined in the text before being used and once defined must be used consistently – there are too many of these to enumerate, e.g. NIHR, G-I-N and PPIE in the abstract; PPIE, QMUL, CMU, HTN and many others in the in manuscript.	Corrected.
I have concerns regarding statements such as "...reach absolute truth" - what is meant by this? How do you define "absolute truth"? (p5 of 63)	Agree. We have removed this term.
The authors must clarify what work has already been undertaken (e.g. to develop an initial program theory) and what is being proposed in this protocol – there appears to be considerable overlap. Has the clinical outcomes trial already begun and if so, what stage has been reached?	We have added details of the IPT and further details of the trial. The implementation of the intervention has started, but not data collection has begun on the realist evaluation. The only activity which has been completed is development of the IPT.
Greater clarity is needed with regards to the proposed methods e.g. what will you do and how, to identify the CMOs – the outcomes are described but the methods for identifying and refining contexts and mechanisms need to be more clearly described and logically organised to adhere to the RAMESES II reporting guidelines and realist methodologies and approaches.	We have revised the manuscript to re-order the data collection and analysis sections to clarify how the mixed methods data will be integrated to develop realist theory
Please describe the methods more precisely – for example: how many consultations will be performed, with which stakeholders and at what time points in the implementation. How exactly will the information from consultations be used to develop the CMOs? What validation will be undertaken with whom and by what means?	We have revised the manuscript to re-order the data collection and analysis sections to clarify how the mixed methods data will be integrated to develop realist theory
What is the definition of urbanity? How will it be measured? What are the categories - is it just urban and rural? – how are these defined in the Thai context?	We have removed the urbanity due to challenges with classification.
An economic evaluation is mentioned without providing any details (p12 of 63). Please clearly articulate the relevance of the economic evaluation to the realist evaluation.	We have now clarified that the realist evaluation will inform scenario analyses in the economic analysis linking level of intervention implementation with trial outcomes and subsequent long-term outcomes and cost-effectiveness.
There is an assertion that "programmes achieved success… " however, no evidence has been provided of this success (p22 of 63).	We have provided the necessary references showing the success of the programmes and plans for future scale-up.
Figures and tables:<br>Some of the figures are illegible e.g. Figure 4;<br>One figure has captions in Thai script – this is not appropriate for an international audience; The figures need footnotes to define the many acronyms used.<br>Do the authors have permissions to reproduce Figure 2 - the figure seems to have been copied an existing publication.<br>It's not clear who "commissioners" refers to in Figure 2.	We have replaced the images as requested. We have permissions to use the image in Figure 2 as the authors of this paper are affiliated with the Clinical Effectiveness Group.
References:<br>The references are not consistently formatted (especially references to online documents or websites) and there are a number of other problems e.g. why are you	We have now corrected the references as requested.

referring to a pre-print from 2022? (ref 5) – either refer to the published paper or choose another reference, there are incomplete references e.g. ref 18.	
